# Rates of bronchopulmonary dysplasia in very low birth weight neonates: a systematic review and meta-analysis

**DOI:** 10.1186/s12931-024-02850-x

**Published:** 2024-05-24

**Authors:** Alvaro Moreira, Michelle Noronha, Jooby Joy, Noah Bierwirth, Aina Tarriela, Aliha Naqvi, Sarah Zoretic, Maxwell Jones, Ali Marotta, Taylor Valadie, Jonathan Brick, Caitlyn Winter, Melissa Porter, Isabelle Decker, Matteo Bruschettini, Sunil K. Ahuja

**Affiliations:** 1 Department of Pediatrics, Division of Neonatology, University of Texas Health Science Center at San Antonio, San Antonio, TX 78229-3900 USA; 2 University of Texas Rio Grande Valley School of Medicine, Edinburg, TX USA; 3 Department of Pediatrics, University of Texas Southwestern, Dallas, TX USA; 4 Department of Pediatrics, Lund University, Lund, Sweden; 5 Veterans Administration Research Center for AIDS and HIV-1 Infection and Center for Personalized Medicine, South Texas Veterans Health Care System, San Antonio, TX USA; 6 Veterans Administration Center for Personalized Medicine, South Texas Veterans Health Care System, San Antonio, TX USA; 7 The Foundation for Advancing Veterans’ Health Research, South Texas Veterans Health Care System, San Antonio, TX USA; 8 Department of Microbiology, Immunology & Molecular Genetics, University of Texas Health Science Center at San Antonio, San Antonio, TX USA; 9 Department of Medicine, University of Texas Health Science Center at San Antonio, San Antonio, TX USA; 10 Department of Biochemistry and Structural Biology, University of Texas Health Science Center at San Antonio, San Antonio, TX USA

**Keywords:** Bronchopulmonary dysplasia, Chronic lung disease, Rates, Prevalence, Meta-analysis

## Abstract

**Importance:**

Large-scale estimates of bronchopulmonary dysplasia (BPD) are warranted for adequate prevention and treatment. However, systematic approaches to ascertain rates of BPD are lacking.

**Objective:**

To conduct a systematic review and meta-analysis to assess the prevalence of BPD in very low birth weight (≤ 1,500 g) or very low gestational age (< 32 weeks) neonates.

**Data sources:**

A search of MEDLINE from January 1990 until September 2019 using search terms related to BPD and prevalence was performed.

**Study selection:**

Randomized controlled trials and observational studies evaluating rates of BPD in very low birth weight or very low gestational age infants were eligible. Included studies defined BPD as positive pressure ventilation or oxygen requirement at 28 days (BPD28) or at 36 weeks postmenstrual age (BPD36).

**Data extraction and synthesis:**

Two reviewers independently conducted all stages of the review. Random-effects meta-analysis was used to calculate the pooled prevalence. Subgroup analyses included gestational age group, birth weight group, setting, study period, continent, and gross domestic product. Sensitivity analyses were performed to reduce study heterogeneity.

**Main outcomes and measures:**

Prevalence of BPD defined as BPD28, BPD36, and by subgroups.

**Results:**

A total of 105 articles or databases and 780,936 patients were included in this review. The pooled prevalence was 35% (95% CI, 28-42%) for BPD28 (*n* = 26 datasets, 132,247 neonates), and 21% (95% CI, 19-24%) for BPD36 (*n* = 70 studies, 672,769 neonates). In subgroup meta-analyses, birth weight category, gestational age category, and continent were strong drivers of the pooled prevalence of BPD.

**Conclusions and relevance:**

This study provides a global estimation of BPD prevalence in very low birth weight/low gestation neonates.

**Supplementary Information:**

The online version contains supplementary material available at 10.1186/s12931-024-02850-x.

## Introduction

Bronchopulmonary dysplasia (BPD), characterized as an arrest of lung growth and development, is an important cause of morbidity and mortality in very preterm newborns [1]. While interventions in neonatal care have led to survival of smaller and younger neonates, therapies for BPD are still limited [2]. Therefore, there is an urgent need for early prediction of BPD and implementation of strategies and therapies that can attenuate disease progression. To accomplish such endeavors, we must first ascertain large-scale estimates of BPD and its global impact over time. In doing so, the effect of interventions and progress towards reducing rates of BPD can be more readily measured. Valid and consistent estimates of the prevalence of BPD around the globe are largely lacking.

A previous study estimated global rates of BPD; however, the definition of BPD was not determined *a priori* and the estimation was reported as a set of ranges per country as opposed to a pooled rate [3]. Challenges to estimating comprehensive rates of BPD include the varying definitions (e.g., 28 day versus 36 week assessment [4, 5]), as well as the heterogeneous inclusion criteria of preterm neonates in studies (e.g., gestational-based inclusion compared to birth weight-based parameters or a combination of both). To overcome these barriers, we sought to conduct a systematic review and meta-analysis that would: (i) estimate global trends in the prevalence of BPD, (ii) examine temporal changes in BPD rates, and (iii) stratify BPD rates according to definition, birth weight, gestational age, setting, continent, and gross domestic product (GDP).

## Methods

We conducted a systematic review and meta-analysis according to recommendations from the Cochrane Handbook for Systematic Reviews of Interventions and adhered to the Preferred Reporting Items for Systematic Reviews and Meta-Analyses (PRISMA) criteria [6]. A protocol of this review was not registered.

### Search strategy

Two investigators (A.M. and M.N.) systematically searched MEDLINE from January 1990 to September 30th, 2019. Search terms included (*bronchopulmonary dysplasia* OR *chronic lung disease)* AND a list of each country. Articles were filtered to include children between the age range of birth and 1 month post-term, no limits were placed on language, and refined to remove review articles. Furthermore, review of references from included studies was performed to supplement our initial search. The full search strategy is presented in eMethods 1 in the Supplement. Lastly, we reviewed all the population-based articles from a systematic review by Siffel et al. [3] wherein they examined global rates of BPD. To enhance the comprehensiveness of our investigation, we integrated national registries from countries that were publicly available documenting outcomes related to BPD.

### Study selection

Two groups of investigators (group 1: A.T. and M.N.; group 2: A.N. and A.M) independently reviewed the titles and abstracts of all citations to determine suitability for inclusion. This was followed by independent review of the full-text articles to confirm eligibility. A third author (S.Z.) resolved any disagreements. Studies were included if they were international or national level (e.g., population-based) studies reporting rates of BPD from 1990 to 2019. The search was initiated from 1990, as this marks the time when surfactant therapy became increasingly standard of care in neonatal centers [7]. The end date was chosen as 2019 to exclude publications using the newest definition for BPD [8]. We included data for all neonates at risk for BPD with confirmed diagnosis occurring in the hospital or prior to discharge. Studies with inclusion criteria of male and female neonates with a birth weight of less than or equal to 1,500 g or a gestational age of less than 32 weeks were included. Due to limited availability of granular patient-level data in the included studies, mortality rates for each study were collected. Case reports, editorials, and commentaries were excluded.

### Data extraction

Two sets of authors (A.T. and M.N.; A.N. and A.M.) independently collected study details. Two authors (J.J. and S.Z.) independently verified the accuracy of collated information. Inconsistencies were discussed among a panel of at least four investigators. Study specifics included country, BPD definition, BPD rates, total number of neonates in the study, years of observation, inclusion criteria, and study design. Articles and standardized data collection sheets were maintained in Google Drive folders. GetData Graph Digitizer version 2.26.0.20 was used to collect values from figures when mortality data was not described in the article text.

### Risk of bias

The risk of bias was judged in a binary fashion (e.g., yes = 1 or no = 0). We assessed the risk of bias for observational studies according to the Newcastle-Ottawa Quality Assessment Scale in three dimensions, selection, comparability, and outcome. The score for observational studies ranged from 0 to 8, representing bias risk for each article. Studies were defined as having a high risk of bias if the total score was five or lower, moderate risk of bias if the score was between five and six, and low bias if the total summed to greater than seven. We assessed the risk of bias for controlled studies according to the Cochrane Risk of Bias Tool using seven dimensions, selection bias (including random sequence generation and allocation concealment), reporting bias, other bias, performance bias, detection bias, and attrition bias. The score for randomized controlled studies ranged from 0 to 7, representing bias risk for each article.

### Definitions and outcomes

*A priori*, BPD was defined by two categories: (i) BPD28- supplemental oxygen or positive pressure ventilation at 28 days of life, and (ii) BPD36- supplemental oxygen or positive pressure ventilation at 36 weeks postmenstrual age. The pooled prevalence of BPD is presented as forest plots for BPD28 and BPD36. If the study stratified patient numbers by both definitions, we included both to each pooled rate. When articles overlapped in time period for a particular country, the articles with more comprehensive data were selected for inclusion. Prespecified subgroup analyses included birth weight categories, gestational age, years, setting, continent, and gross domestic product (GDP). Precisely, gestational age was divided into extremely low gestational age (ELGA) (≤ 28 weeks) vs. very low gestational age (VLGA) (< 32 weeks), while study setting was stratified into international or national. Study years were binned into three decades: 1990–1999, 2000–2009, 2010–2019. This approach was used to explore temporal changes in BPD. The year 1990 was used as the time of inception as the late 1980s and early 1990s is when clinical trials for surfactant use demonstrated efficacy in the care of preterm neonates with respiratory distress syndrome. Birth weight was sorted into extremely low birth weight (ELBW) (*≤* 1,000 g), very low birth weight (VLBW) (≤ 1,500 g), and modifications of these terms (e.g., 501–750 g, 751–1000 g, 1001–1250 g, and 1251–1500 g). To clarify, the subgroup analysis by birth weight of 1000 g was conducted by categorizing studies based on the specified birth weight ranges. Specifically, studies were included in this subgroup analysis if they reported data on all infants falling within the designated birth weight range of interest and not average birthweight reported for a cohort.

### Statistical analysis

The primary outcome was expressed using direct proportions (PR) with a 95% confidence interval (CI) following Freeman-Tukey double arc-sine transformation of the raw data [9]. Expecting high heterogeneity, defined as an *I*^*2*^#x2009;> 50%, all analyses used a DerSimonian–Laird estimate with a random-effects meta-analysis model. The presence of publication bias was evaluated qualitatively using funnel plots and quantitatively conducing Egger’s linear regression test. At least ten studies were needed to perform subgroup analyses. All statistical analyses were performed using R version 4.1.0.

## Results

### Identification of Eligible studies

**Fig. 1:**
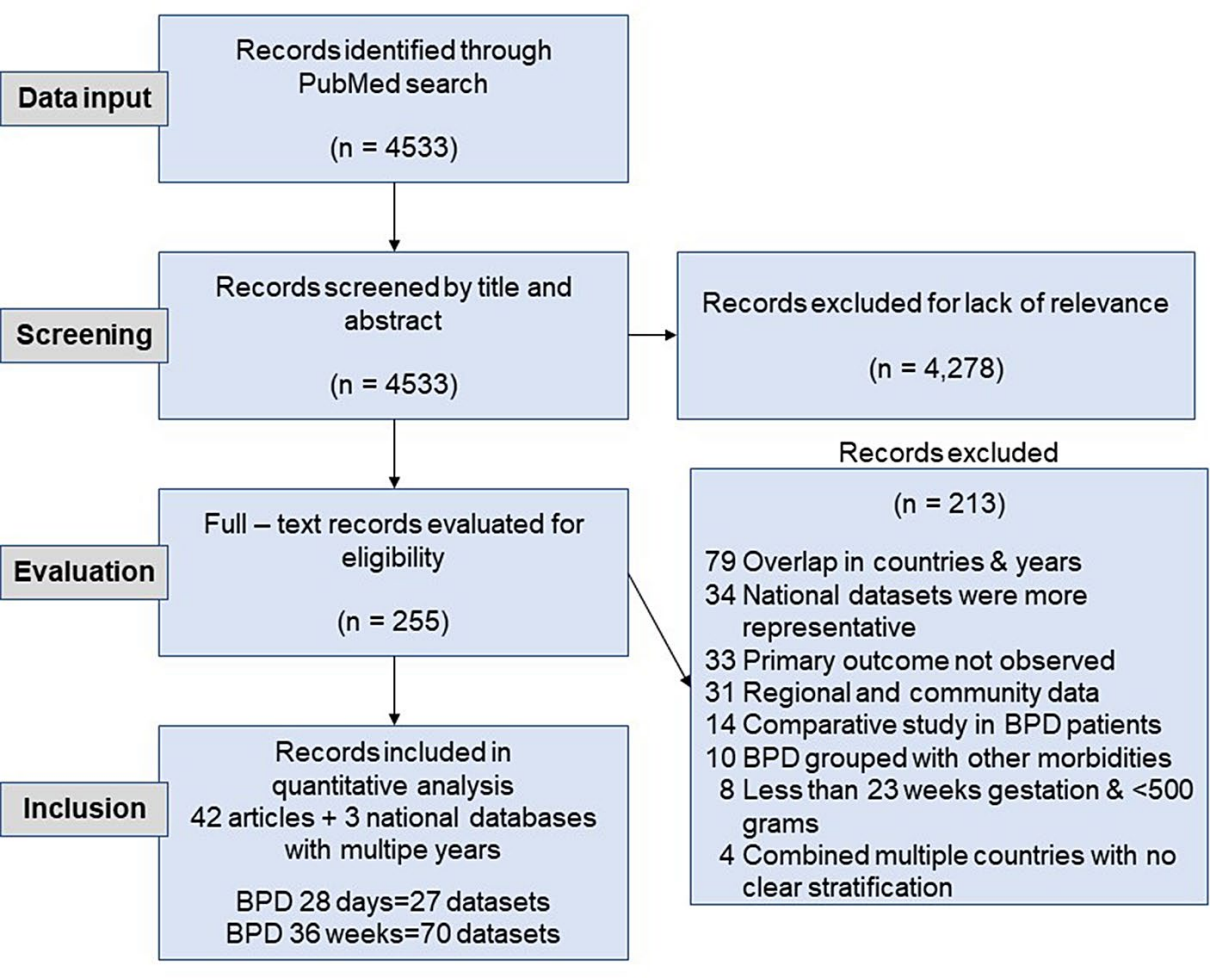
Life satisfaction scores at age 30. 12 = highest possible score. 3 = Lowest possible score

Our search yielded 4582 records, of which 2318 were reviewed in full. After applying the eligibility criteria, a total of 42 were included in this review. We also identified three publicly available national datasets: Australian and New Zealand Neonatal Network, Canadian Neonatal Network, and Neonatal Research Network of Japan Database. Meta-analyses were performed on all studies and databases, moving forward now referred to as datasets. In sum, a combination of 74 datasets comprised the analysis for BPD28 and BPD26 as well as their subgroup analyses. The flow diagram of selected articles is shown in Fig. 1.

**Figure 1 PRISMA flowchart of literature identification and study selection.**

### Study characteristics

Table 1 provides detailed characteristics of the included articles. All the chosen articles were based on cohort investigations and on the two predetermined BPD definitions: BPD28 and BPD36. The most commonly used definition for BPD was BPD36. Thirty countries were represented in the studies, and the countries that produced the most data were Australia and New Zealand (*n* = 24/70 datasets, 34.3%). A total of 672,769 patients were included in this review. Twenty-six out of the 70 datasets (37.1%) in BPD36 were published from 2010 onwards.

**Table 1:** Study characteristics of included articles

Article No.	Author	Country	Data years	Number of BPD patients	Number of patients	Definition of BPD	Type of representativeness
1	Álvarez-Fuente	Spain	2013	917	2628	O2 at 28 days	National
2	Ancel	France	2011	251	4308	O2 at 36 weeks	National
3	Bevilacqua	Italy	1996	47	268	O2 at 28 days	National
4	Bonamy	Belgium, Denmark, Estonia, France, Germany, Italy, Netherlands, Poland, Portugal, UK, Sweden	2011–2012	94	1951	O2 at 36 weeks	Multinational
5	Chen	Switzerland	2000–2012	840	8899	O2 at 36 weeks	National
6	Choi	Korea	2007–2008	1191; 685	3841	O2 at 28 days and 36 weeks	National
7	Fanaroff	United States	1991–1992	1284; 771	4279	O2 at 28 days and 36 weeks	National
8	Fanaroff	United States	1997–2002	5151; 4224	18,153	O2 at 28 days and 36 weeks	National
9	Fortmann	Germany	2009–2015	2118	13,343	O2 at 36 weeks	National
10	Gortner	Belgium, Denmark, France, Germany, Italy, Poland, Portugal, UK	2003	285	1603	O2 at 36 weeks	Multinational
11	Grandi	Argentina, Brazil, Chile, Paraguay, Peru, Uruguay	2001–2010	2768; 1861	11,332	O2 at 28 days and 36 weeks	Multinational
12	Grisaru-Granovsky	Israel	1995–2007	1152	7913	O2 at 28 days	National
13	Guimarães	Portugal	2004–2006	33	256	O2 at 36 weeks	National
14	Guinsberg	Brazil	2012–2013	497	2646	O2 at 36 weeks	National
15	Hentschel	Switzerland	1996&2000	184	1236	O2 at 36 weeks	National
16	Horbar	United States	2005–2014	95,260	327,840	O2 at 36 weeks	National
17	Kamper	Denmark	1994–1995	43	269	O2 at 36 weeks	National
18	Koc	Turkey	2016–2017	800	3381	O2 at 36 weeks	National
19	Kong	China	2013–2014	200	1749	O2 at 28 days	National
20	Kusuda	Japan	2003	601	2145	O2 at 36 weeks	National
21	Lee	Canada	1996–1997	1011	3494	O2 at 36 weeks	National
22	Lee	South Korea	2013–2016	2440	7441	O2 at 36 weeks	National
23	Lemons	United States	1995–1996	1598; 1021	4438	O2 at 28 days and 36 weeks	National
24	Marret	France	1997–2002	89	1638	O2 at 28 days	National
25	Murphy	Ireland	2004–2007	529	2889	O2 at 36 weeks	Multinational
26	Persson	Canada, Finland, Israel, Italy, Japan, Sweden, UK	2007–2015	19,155	76,360	O2 at 36 weeks	Multinational
27	Qiu	Canada	2005	717	3242	O2 at 36 weeks	National
28	Rodrigo	Spain	2002–2006; 2007–2011	369	2485	O2 at 36 weeks	National
29	Rutkowska	Poland	2014–2015	319	707	O2 at 36 weeks	National
30	Sasaki	Japan	2003–2008	5354	15,765	O2 at 28 days	National
31	Skromme	Norway	1999–2000	165	372	O2 at 36 weeks	National
32	Stensvold	Norway	2013–2014	78	185	O2 at 36 weeks	National
33	Stevenson	United States	1993–1994	1424; 260	4593; 999	O2 at 28 days and 36 weeks	National
34	Su	Taiwan	1997–2011	3193	10,479	O2 at 36 weeks	National
35	Toome	Estonia	2002–2003; 2007–2008	115	601	O2 at 36 weeks	National
36	Tsou	Taiwan	1997–2011	113	485	O2 at 28 days	National
37	Tyson	United States	1996–1997	534	807	O2 at 2836 weeks	National
38	Vanhaesebrouck	Belgium	1999–2000	143; 78	175	O2 at 28 days and 36 weeks	National
39	Waal	Netherlands	2007	35	144	O2 at 36 weeks	National
40	Walsh	United States and Canada	2001&2003	21,739	75,974	O2 at 36 weeks	Multinational
41	Watson	England	2009–2011	6551	20,554	O2 at 28 days	National
42	Weber	Austria	1999–2001	95	321	O2 at 36 weeks	National
43	Report of the Australian and New Zealand Neonatal Network	Australia and New Zealand	1995	512	2863	O2 at 36 weeks	Multinational
44	Report of the Australian and New Zealand Neonatal Network	Australia and New Zealand	1996	501	2964	O2 at 36 weeks	Multinational
45	Report of the Australian and New Zealand Neonatal Network	Australia and New Zealand	1997	549	2979	O2 at 36 weeks	Multinational
46	Report of the Australian and New Zealand Neonatal Network	Australia and New Zealand	1998	577	3084	O2 at 36 weeks	Multinational
47	Report of the Australian and New Zealand Neonatal Network	Australia and New Zealand	1999	643	2883	O2 at 36 weeks	Multinational
48	Report of the Australian and New Zealand Neonatal Network	Australia and New Zealand	2000	752	2972	O2 at 36 weeks	Multinational
49	Report of the Australian and New Zealand Neonatal Network	Australia and New Zealand	2001	740	2924	O2 at 36 weeks	Multinational
50	Report of the Au3349stralian and New Zealand Neonatal Network	Australia and New Zealand	2002	692	2944	O2 at 36 weeks	Multinational
51	Report of the Australian and New Zealand Neonatal Network	Australia and New Zealand	2003	621	2607	O2 at 36 weeks	Multinational
52	Report of the Australian and New Zealand Neonatal Network	Australia and New Zealand	2004	673	3204	O2 at 36 weeks	Multinational
53	Report of the Australian and New Zealand Neonatal Network	Australia and New Zealand	2005	623	3349	O2 at 36 weeks	Multinational
54	Report of the Australian and New Zealand Neonatal Network	Australia and New Zealand	2006	476	3084	O2 at 36 weeks	Multinational
55	Report of the Australian and New Zealand Neonatal Network	Australia and New Zealand	2007	585	3439	O2 at 36 weeks	Multinational
56	Report of the Australian and New Zealand Neonatal Network	Australia and New Zealand	2008	624	3666	O2 at 36 weeks	Multinational
57	Report of the Australian and New Zealand Neonatal Network	Australia and New Zealand	2009	634	3552	O2 at 36 weeks	Multinational
58	Report of the Australian and New Zealand Neonatal Network	Australia and New Zealand	2010	720	3273	O2 at 36 weeks	Multinational
59	Report of the Australian and New Zealand Neonatal Network	Australia and New Zealand	2011	1574;747	3536;3736	O2 at 28 days and 36 weeks	Multinational
60	Report of the Australian and New Zealand Neonatal Network	Australia and New Zealand	2012	1067;782	3348;3520	O2 at 28 days and 36 weeks	Multinational
61	Report of the Australian and New Zealand Neonatal Network	Australia and New Zealand	2013	1218;792	2985;3501	O2 at 28 days and 36 weeks	Multinational
62	Report of the Australian and New Zealand Neonatal Network	Australia and New Zealand	2014	1261;871	3058;3615	O2 at 28 days and 36 weeks	Multinational
63	Report of the Australian and New Zealand Neonatal Network	Australia and New Zealand	2015	885	3449	O2 at 36 weeks	Multinational
64	Report of the Australian and New Zealand Neonatal Network	Australia and New Zealand	2016	1698;1028	3385;3610	O2 at 28 days and 36 weeks	Multinational
65	Report of the Australian and New Zealand Neonatal Network	Australia and New Zealand	2017	1728;988	3263;3262	O2 at 28 days and 36 weeks	Multinational
66	Report of the Australian and New Zealand Neonatal Network	Australia and New Zealand	2018	1721;984	3211;3417	O2 at 28 days and 36 weeks	Multinational
67	Canadian Neonatal Network	Canada	2004	175	687	O2 at 36 weeks	National
68	Canadian Neonatal Network	Canada	2006	298	958	O2 at 36 weeks	National
69	Canadian Neonatal Network	Canada	2016	811	1954	O2 at 36 weeks	National
70	Canadian Neonatal Network	Canada	2017	812	1963	O2 at 36 weeks	National
71	Canadian Neonatal Network	Canada	2018	1223	4023	O2 at 36 weeks	National
72	Neonatal Research Network of Japan Database	Japan	2016	1473;727	3982;3030	O2 at 28 days and 36 weeks	National
73	Neonatal Research Network of Japan Database	Japan	2017	1174;718	3354;3262	O2 at 28 days and 36 weeks	National
74	Neonatal Research Network of Japan Database	Japan	2018	1438;873	3507;3010	O2 at 28 days and 36 weeks	National

### Pooled and stratified prevalence of BPD

The pooled prevalence for BPD28 calculated from 27 datasets and 132,424 neonates was 35% (95% CI, 0.28–0.42) using random effects meta-analysis (Fig. 2). For BPD36 (*n* = 70 studies, 672,769 neonates), the pooled prevalence was 21% (95% CI, 0.19–0.24) (Fig. 3). Table 2 depicts the prevalence of BPD28 and BPD36 according to gestational age, birth weight, study period, continent, setting, and GDP (subgroup analysis).

**Figure 2 Pooled prevalence for BPD28. Forest plot demonstrating pooled prevalence for BPD28 and 95% CI with a random-effects meta-analysis model.**

**Figure 3 Pooled prevalence for BPD36. Forest plot demonstrating pooled prevalence for BPD36 and 95% CI with a random-effects meta-analysis model.**

**Table 2:** Rates of BPD28 and BPD36 using subgroup meta-analysis

Variable	No. of Datasets	No. of Cases	No. of Participants	Prevalence (95% CI)	I^2	*p* value
**Global analysis for BPD**						
BPD 28 day	26	42,868	132,247	0.35 (0.28–0.42)	0.99	< 0.01
BPD 36 weeks	70	178,044	672,769	0.21 (0.19–0.24)	0.99	< 0.01
**Subgroup analysis for BPD 28 day**						
*Birthweight*						
<500 g	6	129	131	0.99 (0.97, 1.00)	0.0	0.51
501–750 g	11	6,593	9,263	0.87 (0.75–0.96)	1.00	< 0.01
751–1000 g	11	7,210	12,005	0.74 (0.62–0.84)	1.00	< 0.01
1001–1250 g	11	4,434	13,454	0.41 (0.31–0.52)	1.00	< 0.01
1251–1500 g	11	2,187	16,367	0.16 (0.11–0.22)	0.99	< 0.01
<=1000 g	1	534	807	0.66 (0.63–0.69)	--	--
<=1500 g	7	14,144	47,355	0.32 (0.25–0.41)	1.00	< 0.01
Overall	58	35,231	99,382	0.61 (0.58–0.63)	1.00	< 0.01
*Gestational age*						
ELGA	9	7,119	8,161	0.90 (0.84–0.95)	0.99	< 0.01
VLGA	17	21,237	70,759	0.29 (0.22–0.38)	1.00	< 0.001
Overall	26	28,356	78,920	0.62 (0.56–0.68)	1.00	< 0.001
*Year*						
1990–1999	5	4,887	14,385	0.36 (0.21–0.52)	0.99	< 0.01
2000–2009	2	6,545	19,606	0.33 (0.30–0.35)	0.92	< 0.01
2010–2019	12	15,469	38,006	0.39 (0.32–0.46)	0.99	< 0.001
Overall	19	26,902	71,997	0.34 (0.31–0.36)	0.99	0.22
*Setting*						
National	18	29,833	98,129	0.42 (0.35–0.50)	1.00	< 0.001
Multinational	8	13,035	34,118	0.32 (0.23–0.41)	0.99	< 0.001
Overall	26	42,868	132,247	0.38 (0.33–0.44)	1.00	0.07
*Continent*						
Asia	7	10,943	32,683	0.30 (0.22–0.38)	1.00	< 0.001
Europe	6	8,899	33,176	0.29 (0.10–0.53)	1.00	< 0.001
North America	5	9,991	32,270	0.38 (0.25–0.52)	0.99	< 0.01
Oceania	7	10,267	22,786	0.45 (0.39–0.51)	0.99	< 0.01
South America	1	2,768	11,332	0.24 (0.24–0.25)	--	--
Overall	26	42,868	132,247	0.25 (0.24–0.26)	1.00	< 0.01
*GDP*						
1st quartile (lowest)	3	1,408	8,573	0.39 (0.05–0.81)	0.99	< 0.01
2nd quartile	3	3,682	10,005	0.37 (0.29–0.45)	0.99	< 0.01
3rd quartile	3	6,687	22,460	0.17 (0.05–0.35)	1.00	< 0.01
4th quartile (highest)	3	2,957	10,010	0.25 (0.11–0.42)	1.00	< 0.001
Overall	12	14,734	51,048	0.33 (0.26–0.39)	1.00	0.17
**Subgroup analysis for BPD 36 weeks**						
*Birthweight*						
<500 g	8	285	391	0.71 (0.51–0.87)	0.91	< 0.01
501–750 g	15	3,877	7,330	0.60 (0.51–0.68)	0.99	< 0.01
751–1000 g	15	4,400	10,585	0.43 (0.38–0.49)	0.97	< 0.01
1001–1250 g	15	2,411	11,803	0.22 (0.18–0.26)	0.95	< 0.01
1251–1500 g	15	1,291	14,293	0.10 (0.07–0.13)	0.94	< 0.01
<=1000 g	1	165	372	0.44 (0.39–0.49)	--	--
<=1500 g	13	148,254	534,587	0.24 (0.20–0.27)	1.00	< 0.01
Overall	82	160,683	579,361	0.28 (0.26–0.30)	0.99	< 0.01
*Gestational age*						
ELGA	29	110,462	358,636	0.43 (0.39–0.48)	1.00	< 0.001
VLGA	42	25,199	126,368	0.12 (0.10–0.14)	0.99	< 0.001
Overall	71	135,661	485,004	0.21 (0.19–0.23)	1.00	< 0.01
*Year*						
1990–1999	10	5,888	28,252	0.21 (0.18–0.23)	0.96	< 0.01
2000–2009	23	9,893	48,359	0.20 (0.18–0.22)	0.95	< 0.01
2010–2019	26	17,440	69,244	0.22 (0.16–0.27)	0.99	< 0.001
Overall	59	33,221	145,855	0.20 (0.19–0.22)	0.99	0.85
*Setting*						
National	33	117,903	425,177	0.26 (0.23–0.30)	1.00	< 0.001
Multinational	37	60,662	247,592	0.18 (0.16–0.21)	0.99	< 0.001
Overall	70	178,565	672,769	0.21 (0.19–0.23)	0.99	< 0.01
*Continent*						
Asia	8	10,037	36,589	0.26 (0.22–0.29)	0.98	< 0.01
Europe	21	5,638	37,787	0.16 (0.11–0.22)	0.99	< 0.001
North America	12	124,098	429,851	0.29 (0.25–0.33)	0.99	< 0.01
North America, Europe, Asia	1	19,155	76,360	0.25 (0.25–0.25)	--	--
Oceania	24	16,999	77,897	0.22 (0.20–0.23)	0.97	< 0.01
South America	2	2,358	13,312	0.18 (0.17–0.19)	0.61	0.11
Overall	68	178,285	671,796	0.25 (0.24–0.25)	0.99	< 0.01
*GDP*						
1st quartile (lowest)	6	734	4,867	0.11 (0.05–0.20)	0.98	< 0.01
2nd quartile	5	5,001	23,367	0.19 (0.06–0.38)	1.00	< 0.01
3rd quartile	5	2,558	12,162	0.18 (0.16–0.19)	0.99	< 0.01
4th quartile (highest)	5	4,752	27,569	0.18 (0.10–0.28)	1.00	< 0.01
Overall	21	13,045	67,965	0.17 (0.12–0.21)	0.99	0.61

#### Subgroup analysis for BPD28

When stratified by birth weight, the highest rates of BPD28 were found in infants with lower birth weights: <1000 g (ELBW). For instance, infants in the lowest birth weight stratum (< 500 g) had a BPD28 prevalence of 99% (95% CI, 0.97-1.00), while those in the second-lowest birth weight stratum (501–750 g) had a BPD28 prevalence of 87% (95% CI, 0.75–0.96). The BPD28 prevalence was lowest (16%; 95% CI, 0.11–0.22) in infants with the highest birth weights (1251–1500 g). The prevalence of BPD28 was higher in ELGA versus VLGA neonates (90% vs. 29%). The subgroup analysis of BPD28 by setting showed a higher rate in the national compared to multinational studies, as well as Oceania compared to other continents. Overall, no differences were observed in BPD28 prevalence when stratified by year or GDP.

#### Subgroup analysis for BPD36

The subgroup analysis for prevalence of BPD36 stratified by birth weight was very similar to the BPD28 analysis, in which an upward trend in the prevalence of BPD36 was associated with lower birth weights. For example, the highest prevalence of BPD36 was noted in neonates with a birth weight of less than 1000 g (ELBW). Further stratification of the ELBW neonates revealed BPD36 prevalence rates of 71% (95% CI, 0.51–0.87) and 60% (95% CI, 0.51–0.68) in neonates with birth weights of < 500 g and 501–750 g, respectively. Again, the lowest prevalence of BPD36, 10% (95% CI, 0.07–0.13), was seen in the highest (1251–1500 g) birth weight stratum.

Similar to the findings using the BPD28 definition, prevalence of BPD36 was higher in ELGA neonates (43% *n* = 358,636, versus 12% *n* = 126,368). Prevalence of BPD36 was also higher in national studies. Lastly, BPD36 prevalence again differed when stratified by continent. The highest prevalence was seen in North America at 329% (95% CI, 0.25–0.33). Rates of BPD36 were similar across GDP strata and year.

### Sensitivity analysis and mortality rate

We conducted sensitivity analysis on the prevalence of BPD28 and BPD36 to reduce heterogeneity, defined as an I^2^ ≥ 50%. After keeping only 4 studies, the prevalence of BPD28 was 32% (95% CI, 0.31–0.32; I^2^ = 0%, **eResults 1**). For BPD36, 10 studies remained after filtering for high heterogeneity. The resulting rate of BPD36 was 25% (95% CI, 0.25–0.26; I^2^ = 49%, **eResults 2**). The table in **eResults 3** shows the varying range of mortality rates for each of the studies (range of 0–23.9% with an average rate of 8.1%).

### Risk of bias and publication bias

Forty-two studies were evaluated by the Newcastle-Ottawa Quality Assessment Scale and one study by the Cochrane Risk of Bias Tool. Thirty (74%) of the observational studies had a moderate bias (total score ranging from 5 to 6) (**eTable 1**). The domain that had the most bias pertained to questions regarding follow-up outcomes. Nine studies (21%) had low risk of bias (total score between 7 and 8). The single randomized controlled trial had a risk of bias score of five out of seven. Publication bias was low for BPD28 and BPD36. Plots can be viewed in **eFigures 1, 2**.

## Discussion

Bronchopulmonary dysplasia remains the most common morbidity of prematurity and carries a significant disease burden [10]. Throughout the published literature, BPD displays itself as a disease with significant heterogeneity [11–14]. This is found not only within different “types” of BPD but also within the definition itself; as published data defines it as oxygen at 28 days, 36 weeks or other combinations of factors [15]. Therefore, it is essential to have accurate information for prediction, analysis and treatment. We performed this systematic review and meta-analysis to determine large-scale rates of bronchopulmonary dysplasia, with a subgroup analysis according to two major definitions. To our knowledge this is the largest and most comprehensive study describing BPD prevalence to date.

Our study expands on the 2019 study by Siffel et al. [3] to provide a more complete review of available data. We discovered, reviewed and analyzed data over a 41-year period (versus 11 years), with inclusion of a higher number of studies across more regions. As an additional contrast, we defined BPD (oxygen at 28 days or 36 weeks) and manually extracted data for combined analysis. This allowed us to use pooled data to compare subgroups and pursue further statistical analyses. We were therefore able to provide a more accurate prevalence for each provided outcome, rather than reporting outcomes as a set of ranges from individual studies.

As anticipated, the foremost risk factor for developing BPD was found to be low birth weight, particularly with a weight below 750 g. This trend was evident across both individual subgroup analyses and combined evaluations. Additionally, our observations revealed discrepancies in BPD rates among different gestational age groups, notably between ELGA and VLGA infants. These findings align with existing literature that underscores an inverse association between BPD rates and gestational age/birthweight, further affirming the current understanding in the field [8, 16]. 

We also compared BPD rates across three decades (1990–1999, 2000–2010 and 2010–2020), which showed no difference between the groups across the definitions of BPD. This is found throughout the literature and highlights the difficulty in preventing and treating this disease. Medical advancements in the care of preterm neonates have led to higher survival, especially in the most industrialized nations [17, 18]. This coincides with the survival of more infants with BPD and accounts for much of the similarity of the prevalence across decades. While our study focuses on reporting BPD rates in decade cohorts, it’s essential to acknowledge the limitations inherent in utilizing these broader definitions of BPD. We recognize that the clinical landscape of BPD management may have evolved over the past 30 years, potentially leading to improvements not fully captured by the BPD28 and BPD36 definitions. Our exclusion of studies using the newer BPD definition by Jensen et al. was indeed mentioned in the methods section, but we acknowledge the importance of reiterating this point here for clarity.

While the incidence of BPD exhibits considerable variation among different countries, current evidence indicates minimal disparities in its prevalence across major continents. Numerous studies have explored BPD incidence and associated risk factors in various regions spanning North America, Europe, Asia, and Australia, generally yielding comparable rates. For instance, research by Jain et al. found no significant divergence in BPD incidence among preterm infants across North America, Europe, and Australia [19]. In contrast, our investigation suggests notable differences in BPD rates among regions or continents, particularly with lower rates observed in Europe and South America. However, it’s noteworthy that South America’s data pool was limited to just 1–2 studies. These findings imply that the risk factors and underlying pathophysiology of BPD may not uniformly align across geographical regions, underscoring the imperative for further investigation to elucidate these distinctions. This prompts consideration as to whether disparities in clinical practices might potentially justify these findings.

The Neonatal Research Network (NRN) in the United States has compiled large retrospective analyses of care practice and patient outcomes among extremely premature infants. They have demonstrated that rates of antenatal steroids and surfactant administration have increased, delivery room intubation has decreased [7]. However, the rates of bronchopulmonary dysplasia (BPD36) in their study ranged from 32 to 45%, which is notably higher than the 21% observed in this study. This difference could be attributed to the varying gestational ages included in the studies, as the NRN’s research comprised newborns between 22 and 28 weeks. In comparison, the Chinese Neonatal Network’s cohort of 8,148 preterm neonates had a BPD36 rate of 29.2%, which is higher than our study’s results, again differences most likely due to their inclusion of neonates 31 weeks and younger whereas our study included neonates of ≤ 32 weeks [20]. 

The prevalence of BPD varied depending on the study setting, with national cohorts demonstrating the highest rates for both definitions of BPD. These estimates may be more reliable, as they offer a broader representation across multiple institutions, reducing the impact of outliers and the unique management practices of individual hospitals on the results. Furthermore, many of these national studies employed inclusion criteria that targeted younger gestational ages, further enhancing their robustness. Despite the thought that GDP may have an impact on BPD rates, subgroup analyses based on quartiles of a nation’s GDP showed no differences. One possible explanation for this finding is that other factors beyond GDP, such as access to healthcare and neonatal resources, may play a more significant role.

### Limitations

Despite conducting an extensive data search employing multiple reviewers and diverse search methods, there remains a possibility that certain available studies may have been overlooked. Our findings reveal considerable heterogeneity across all examined outcomes, with many I2 values approaching 1. Despite efforts to minimize this through meticulous data extraction and analysis, the persistence of heterogeneity underscores the importance of cautiously applying the results to specific disease populations. For example, Bonamy et al. reported low BPD rates as it exclusively classified the condition in individuals with the severe form of the disease. In an attempt to mitigate the observed heterogeneity, we conducted a sensitivity analysis, which yielded rates comparable to those obtained in the initial analysis characterized by high heterogeneity.

Another constraint stems from the limited granularity of the original datasets, owing to the diverse definitions of BPD and the myriad ways in which data can be presented. This limitation restricts our ability to conduct more sophisticated statistical analyses and may lead to unequal weighting of studies where data accessibility varies. Additionally, there is a notable disparity in the amount of data available for some regions, notably North America, Oceania, and Europe, compared to other global populations. It would have been ideal to gather data as comprehensive as that publicly available from Australia and New Zealand, Canada, and Japan. Moreover, handling mortality data was a significant challenge in our analysis. We encountered variations among studies, where some solely included survivors while others reported mortality rates without adjusting them in their BPD rates. Some observed rates may have been exceptionally low, especially if their mortality rates were high. We were unable to solely include survivors due to variations in study methodologies, with some studies including only survivors while others encompassed all patients in their denominator for BPD, regardless of neonatal mortality. Adapting our analysis to account for this disparity without access to patient-level data limited our analyses. To address this limitation, we included mortality rates in the supplementary materials. This allows for transparency regarding the impact of mortality on our findings and provides additional context for interpreting the results. While we hypothesized differences in pathophysiology as a possible cause for national differences, it is essential to acknowledge other potential factors that may influence BPD rates, such as variations in reporting practices, gestational age and birth weight distributions, and early mortality rates. These factors could contribute to the observed regional differences in BPD rates and warrant further investigation. Also, differences in the sophistication of medical treatment across regions impacts survival and eventual diagnosis of BPD, all of which affect overall outcomes and generalizability.

## Conclusions

To conclude, this large systematic review and meta-analysis shows that despite advancements, the prevalence of bronchopulmonary dysplasia has remained consistent through decades and is a significant burden across populations. The data generated from this study could serve as baseline rates for future research and could help guide the development of bundled care strategies aimed at decreasing BPD rates [21]. Ultimately, a greater understanding of modifiable factors that contribute to BPD development is critical to improving outcomes and reducing the burden of this disease.

### Electronic supplementary material

Below is the link to the electronic supplementary material.

Supplementary Material 1

Supplementary Material 2

Supplementary Material 3

Supplementary Material 4

Supplementary Material 5

Supplementary Material 6

Supplementary Material 7

Supplementary Material 8

Supplementary Material 9

Supplementary Material 10

## Data Availability

All data generated or analyzed during this study are included in this published article and its supplementary information.

## Declarations

### Originality of content

All information and materials in the manuscript are original.

## Abbreviations

BPD: bronchopulmonary dysplasia
GDP: gross domestic product
ELGA: extremely low gestational age
VLGA: very low gestational age
ELBW: extremely low birth weight
VLBW: very low birth weight
CI: confidence interval
NRN: Neonatal Research Network
