# Supplementary material for: Rates of bronchopulmonary dysplasia in very low birth weight neonates: a systematic review and meta-analysis
Source: Respir Res. 2024 May 24;25:219. doi: 10.1186/s12931-024-02850-x (PMC11127341; doi:10.1186/s12931-024-02850-x)
Supplement: Supplementary file 1 — Supplementary Material 1 [file 12931_2024_2850_MOESM1_ESM.docx]

**eMethods 1.** **PubMed search**

Search conducted on May 29^th^, 2020. N=4533 studies.

Search: ((Afghanistan OR Albania OR Algeria OR Andorra OR Angola OR Antigua and Barbuda OR Argentina OR Armenia OR Australia OR Austria OR Azerbaijan OR Bahamas OR Bahrain OR Bangladesh OR Barbados OR Belarus OR Belgium OR Belize OR Benin OR Bhutan OR Bolivia OR Bosnia OR Herzegovina OR Botswana OR Brazil OR Brunei OR Bulgaria OR Burkina Faso OR Burundi OR Cabo Verde OR Cambodia OR Cameroon OR Canada OR Central African Republic OR Chad OR Chile OR China OR Colombia OR Comoros OR Congo OR Costa Rica OR Cote d'Ivoire OR Croatia OR Cuba OR Cyprus OR Czechia OR Denmark OR Djibouti OR Dominica OR Dominican Republic OR Ecuador OR Egypt OR El Salvador OR Equatorial Guinea OR Eritrea OR Estonia OR Eswatini OR Swaziland OR Ethiopia OR Fiji OR Finland OR France OR Gabon OR Gambia OR Georgia OR Germany OR Ghana OR Greece OR Grenada OR Guatemala OR Guinea OR Guinea-Bissau OR Guyana OR Haiti OR Honduras OR Hungary OR Iceland OR India OR Indonesia OR Iran OR Iraq OR Ireland OR Israel OR Italy OR Jamaica OR Japan OR Jordan OR Kazakhstan OR Kenya OR Kiribati OR Kosovo OR Kuwait OR Kyrgyzstan OR Laos OR Latvia OR Lebanon OR Lesotho OR Liberia OR Libya OR Liechtenstein OR Lithuania OR Luxembourg OR Madagascar OR Malawi OR Malaysia OR Maldives OR Mali OR Malta OR Marshall Islands OR Mauritania OR Mauritius OR Mexico OR Micronesia OR Moldova OR Monaco OR Mongolia OR Montenegro OR Morocco OR Mozambique OR Myanmar OR Burma OR Namibia OR Nauru OR Nepal OR Netherlands OR New Zealand OR Nicaragua OR Niger OR Nigeria OR North Korea OR North Macedonia OR Macedonia OR Norway OR Oman OR Pakistan OR Palau OR Palestine OR Panama OR Papua New Guinea OR Paraguay OR Peru OR Philippines OR Poland OR Portugal OR Qatar OR Romania OR Russia OR Rwanda OR Saint Kitts OR Nevis OR Saint Lucia OR Saint Vincent OR Grenadines OR Samoa OR San Marino OR Sao Tome OR Principe OR Saudi Arabia OR Senegal OR Serbia OR Seychelles OR Sierra Leone OR Singapore OR Slovakia OR Slovenia OR Solomon Islands OR Somalia OR South Africa OR South Korea OR South Sudan OR Spain OR Sri Lanka OR Sudan OR Suriname OR Sweden OR Switzerland OR Syria OR Taiwan OR Tajikistan OR Tanzania OR Thailand OR Timor-Leste OR Togo OR Tonga OR Trinidad OR Tobago OR Tunisia OR Turkey OR Turkmenistan OR Tuvalu OR Uganda OR Ukraine OR United Arab Emirates OR United Kingdom OR United States OR Uruguay OR Uzbekistan OR Vanuatu OR Vatican City OR Holy See OR Venezuela OR Vietnam OR Yemen OR Zambia OR Zimbabwe)) AND ((Bronchopulmonary dysplasia OR Chronic lung disease) NOT (review)) Filters: Humans, English, Spanish, MEDLINE, Newborn: birth-1 month, from 1990 - 2020 Sort by: Most Recent
