# Supplementary figures and images for: Rates of bronchopulmonary dysplasia in very low birth weight neonates: a systematic review and meta-analysis
Source: Respir Res. 2024 May 24;25:219. doi: 10.1186/s12931-024-02850-x (PMC11127341; doi:10.1186/s12931-024-02850-x)

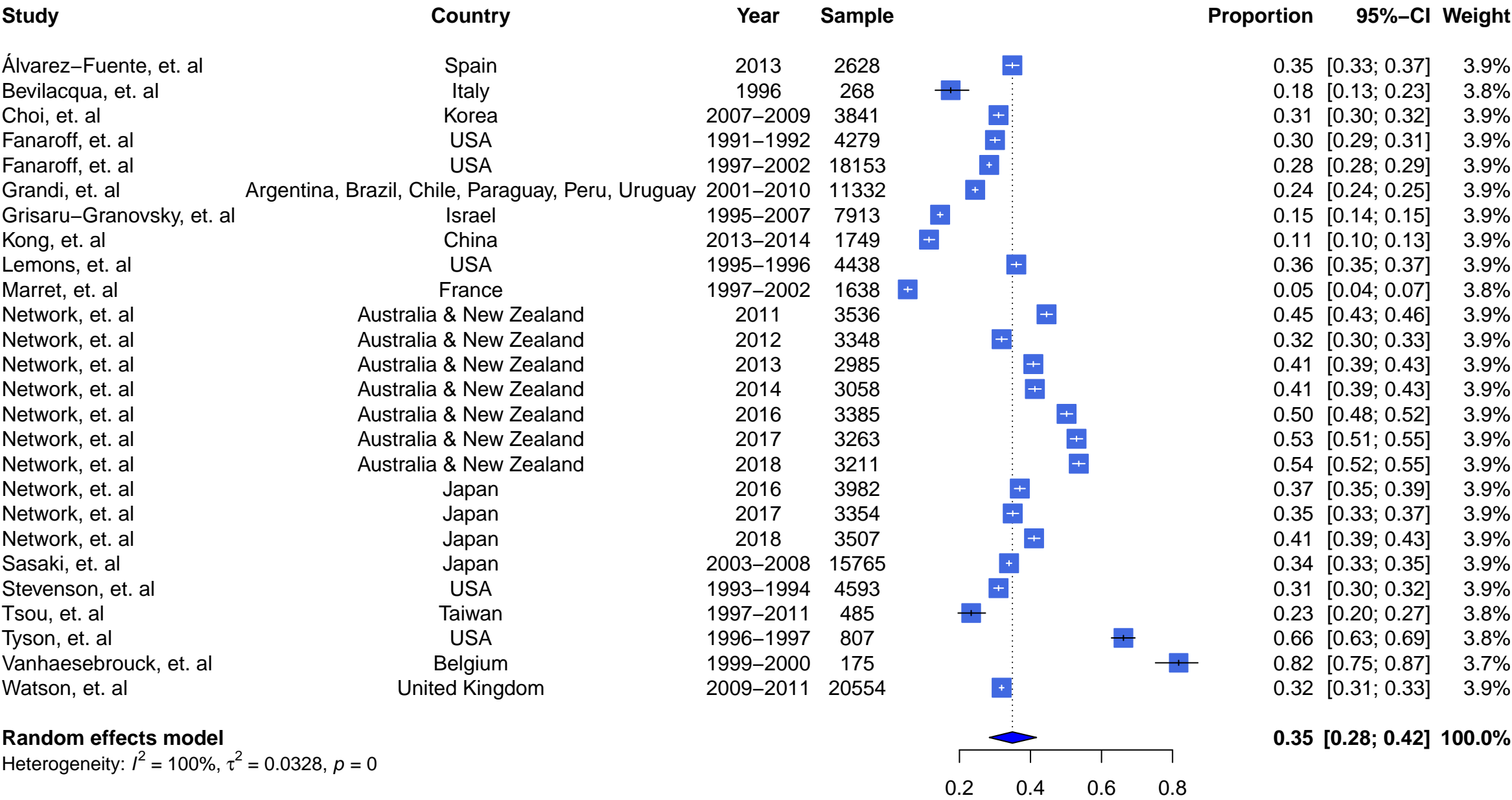

Supplement: Supplementary file 2 — Supplementary Material 2 [file 12931_2024_2850_MOESM2_ESM.pdf]

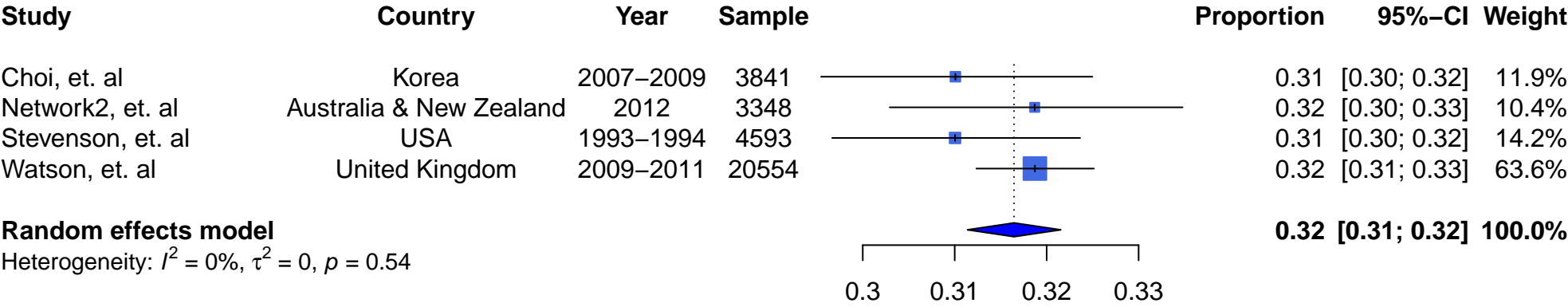

Supplement: Supplementary file 4 — Supplementary Material 4 [file 12931_2024_2850_MOESM4_ESM.pdf]

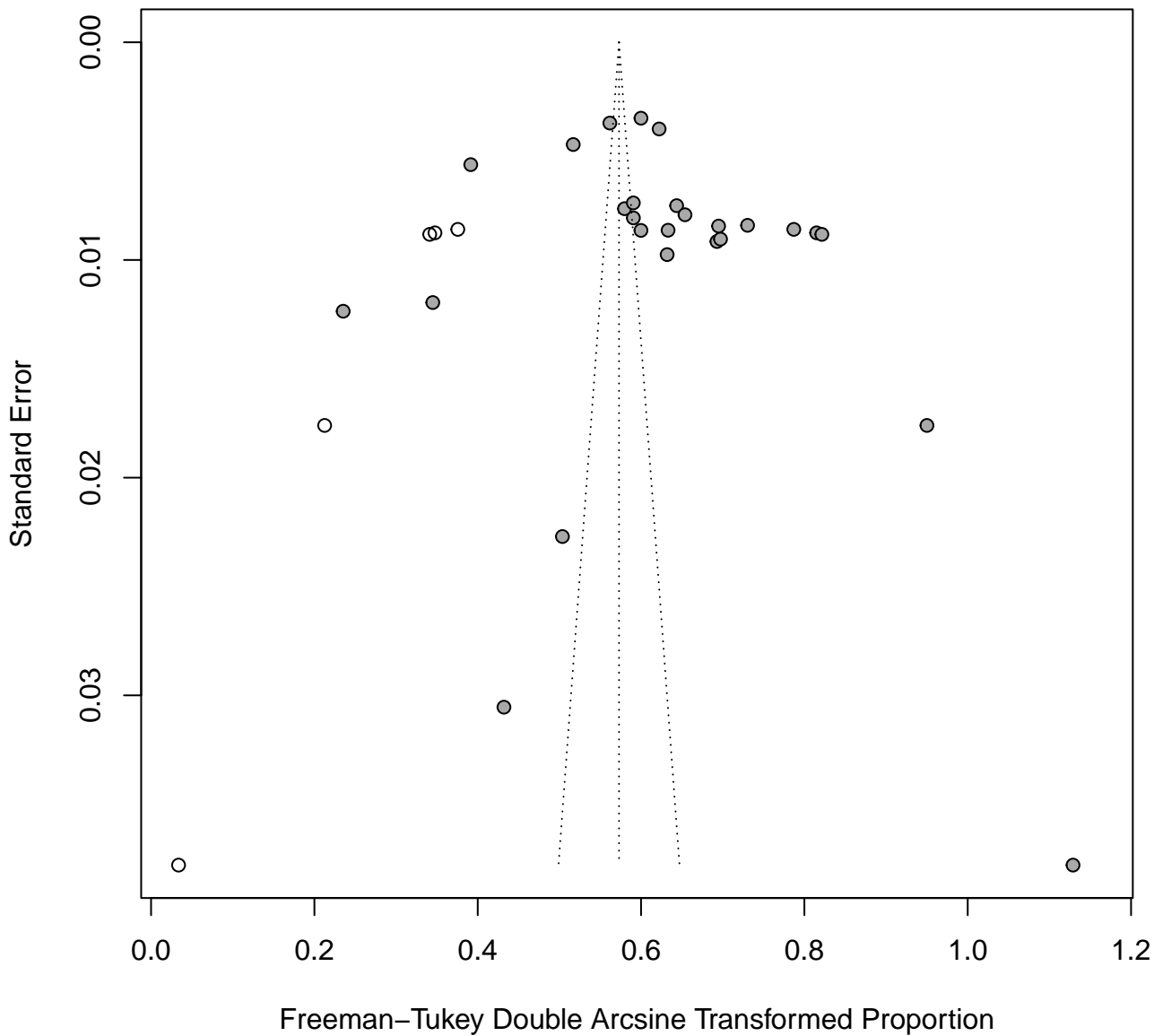

Supplement: Supplementary file 8 — Supplementary Material 8 [file 12931_2024_2850_MOESM8_ESM.pdf]

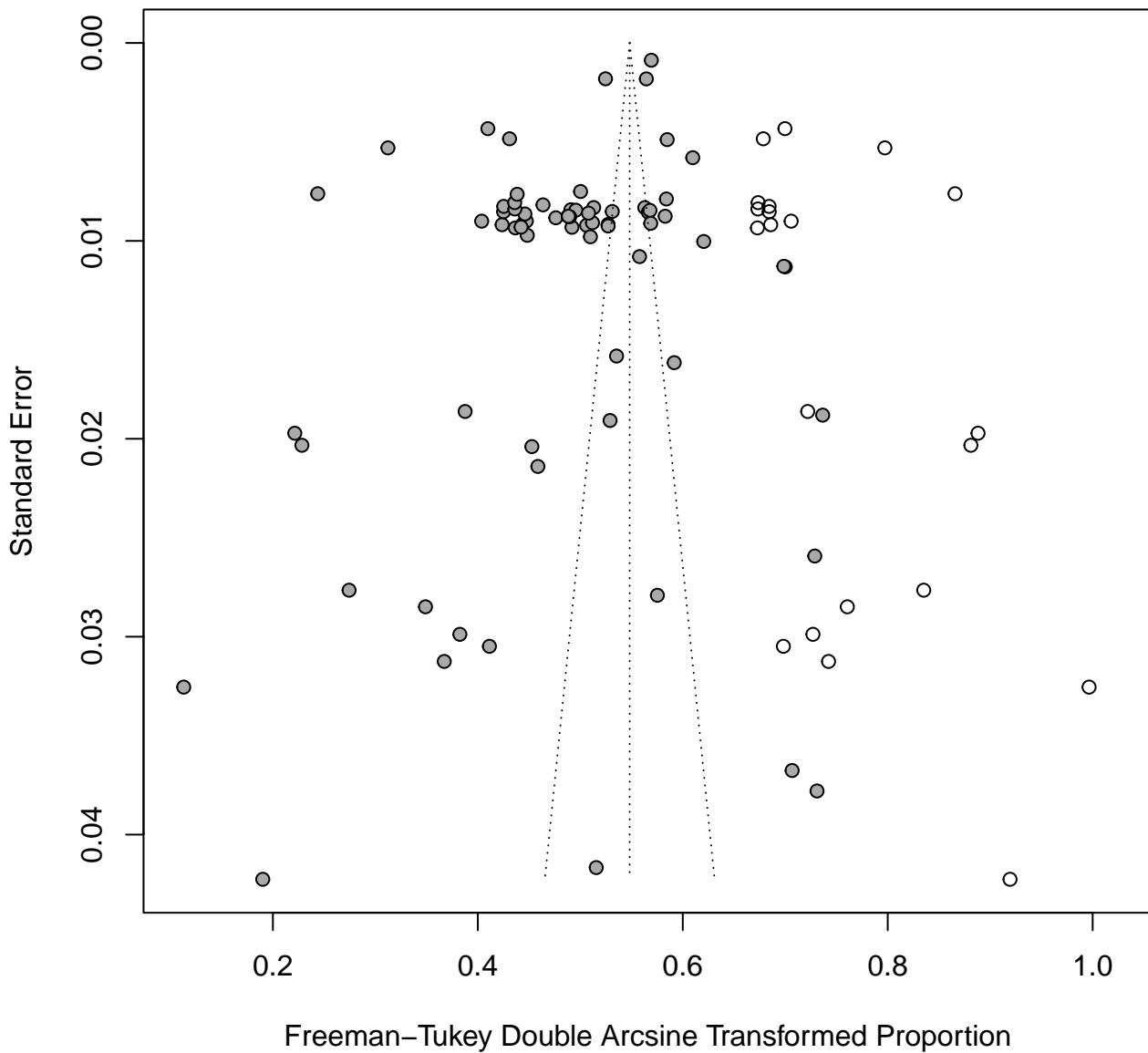

Supplement: Supplementary file 9 — Supplementary Material 9 [file 12931_2024_2850_MOESM9_ESM.pdf]
