# Supplementary material for: Rates of bronchopulmonary dysplasia in very low birth weight neonates: a systematic review and meta-analysis
Source: Respir Res. 2024 May 24;25:219. doi: 10.1186/s12931-024-02850-x (PMC11127341; doi:10.1186/s12931-024-02850-x)

Study	Country	Year	Sample	Proportion	95%−CI	Weight
Ancel, et. al	France	2011	4308	0.06	[0.05; 0.07]	1.4%
Bonamy, et. al	Belgium	2011−2012	643	0.05	[0.03; 0.07]	1.4%
Bonamy, et. al	Estonia	2011−2012	140	0.04	[0.01; 0.07]	1.3%
Bonamy, et. al	Netherlands	2011−2012	327	0.07	[0.05; 0.10]	1.4%
Bonamy, et. al	Poland	2011−2012	236	0.01	[0.00; 0.03]	1.4%
Bonamy, et. al	Portugal	2011−2012	605	0.05	[0.04; 0.07]	1.4%
Chen, et. al	Switzerland	2000−2012	8899	0.09	[0.09; 0.10]	1.5%
Choi, et. al	Korea	2007−2008	3841	0.18	[0.17; 0.19]	1.4%
Fanaroff, et. al	USA	1991−1992	4279	0.18	[0.17; 0.19]	1.4%
Fortmann, et. al	Germany	2009−2015	13343	0.16	[0.15; 0.16]	1.5%
Gortner, et. al	France	2003	721	0.14	[0.12; 0.17]	1.4%
Gortner, et. al	Germany	2003	546	0.20	[0.16; 0.23]	1.4%
Gortner, et. al	Netherlands	2003	308	0.12	[0.08; 0.16]	1.4%
Gortner, et. al	Poland	2003	280	0.14	[0.10; 0.18]	1.4%
Grandi, et. al	Argentina, Brazil, Chile, Paraguay, Peru, Uruguay	2001−2010	10666	0.17	[0.17; 0.18]	1.5%
Guimarães, et. al	Portugal	2004−2006	256	0.13	[0.09; 0.17]	1.4%
Guinsberg, et. al	Brazil	2012−2013	2646	0.19	[0.17; 0.20]	1.4%
Horbar, et. al	USA	2005−2014	327840	0.29	[0.29; 0.29]	1.5%
Kamper, et. al	Denmark	1994−1996	269	0.16	[0.12; 0.21]	1.4%
Koc, et. al	Turkey	2016−2017	3381	0.24	[0.22; 0.25]	1.4%
Kusuda, et. al	Japan	2003	2145	0.28	[0.26; 0.30]	1.4%
Lee, et. al	Canada	1996−1997	3494	0.29	[0.27; 0.30]	1.4%
Lee, et. al	Korea	2013−2016	7441	0.33	[0.32; 0.34]	1.5%
Lemons, et. al	USA	1995−1996	4438	0.23	[0.22; 0.24]	1.4%
Murphy, et. al	Ireland	2004−2007	2889	0.18	[0.17; 0.20]	1.4%
Network, et. al	Australia & New Zealand	1995	2863	0.18	[0.17; 0.19]	1.4%
Network, et. al	Australia & New Zealand	1996	2964	0.17	[0.16; 0.18]	1.4%
Network, et. al	Australia & New Zealand	1997	2979	0.18	[0.17; 0.20]	1.4%
Network, et. al	Australia & New Zealand	1998	3084	0.19	[0.17; 0.20]	1.4%
Network, et. al	Australia & New Zealand	1999	2883	0.22	[0.21; 0.24]	1.4%
Network, et. al	Australia & New Zealand	2000	2972	0.25	[0.24; 0.27]	1.4%
Network, et. al	Australia & New Zealand	2001	2924	0.25	[0.24; 0.27]	1.4%
Network, et. al	Australia & New Zealand	2002	2944	0.24	[0.22; 0.25]	1.4%
Network, et. al	Australia & New Zealand	2003	2607	0.24	[0.22; 0.25]	1.4%
Network, et. al	Australia & New Zealand	2004	3204	0.21	[0.20; 0.22]	1.4%
Network, et. al	Australia & New Zealand	2005	3349	0.19	[0.17; 0.20]	1.4%
Network, et. al	Australia & New Zealand	2006	3084	0.15	[0.14; 0.17]	1.4%
Network, et. al	Australia & New Zealand	2007	3439	0.17	[0.16; 0.18]	1.4%
Network, et. al	Australia & New Zealand	2008	3666	0.17	[0.16; 0.18]	1.4%
Network, et. al	Australia & New Zealand	2009	3552	0.18	[0.17; 0.19]	1.4%
Network, et. al	Australia & New Zealand	2010	3273	0.22	[0.21; 0.23]	1.4%
Network, et. al	Australia & New Zealand	2011	3736	0.20	[0.19; 0.21]	1.4%
Network, et. al	Australia & New Zealand	2012	3520	0.22	[0.21; 0.24]	1.4%
Network, et. al	Australia & New Zealand	2013	3501	0.23	[0.21; 0.24]	1.4%
Network, et. al	Australia & New Zealand	2014	3615	0.24	[0.23; 0.26]	1.4%
Network, et. al	Australia & New Zealand	2015	3449	0.26	[0.24; 0.27]	1.4%
Network, et. al	Australia & New Zealand	2016	3610	0.28	[0.27; 0.30]	1.4%
Network, et. al	Australia & New Zealand	2017	3262	0.30	[0.29; 0.32]	1.4%
Network, et. al	Australia & New Zealand	2018	3417	0.29	[0.27; 0.30]	1.4%
Network, et. al	Canada	2004	687	0.25	[0.22; 0.29]	1.4%
Network, et. al	Canada	2006	958	0.31	[0.28; 0.34]	1.4%
Network, et. al	Canada	2016	1954	0.42	[0.39; 0.44]	1.4%
Network, et. al	Canada	2017	1963	0.41	[0.39; 0.44]	1.4%
Network, et. al	Canada	2018	4023	0.30	[0.29; 0.32]	1.4%
Network, et. al	Japan	2016	3030	0.24	[0.22; 0.26]	1.4%
Network, et. al	Japan	2017	3262	0.22	[0.21; 0.23]	1.4%
Network, et. al	Japan	2018	3010	0.29	[0.27; 0.31]	1.4%
Persson, et. al	Canada, Finland, Israel, Italy, Japan, Sweden, United Kingdom	2007−2015	76360	0.25	[0.25; 0.25]	1.5%
Qiu, et. al	Canada	2005	3242	0.22	[0.21; 0.24]	1.4%
Rodrigo, et. al	Spain	2007−2011	2485	0.13	[0.12; 0.14]	1.4%
Rutkowska, et. al	Poland	2014−2015	707	0.45	[0.41; 0.49]	1.4%
Skromme, et. al	Norway	1999−2000	372	0.44	[0.39; 0.49]	1.4%
Stensvold, et. al	Norway	2013−2014	185	0.42	[0.35; 0.49]	1.3%
Stevenson, et. al	USA	1993−1994	999	0.26	[0.23; 0.29]	1.4%
Su, et. al	Taiwan	1997−2011	10479	0.30	[0.30; 0.31]	1.5%
Toome, et. al	Estonia	2002−2003; 2007−2008	601	0.19	[0.16; 0.22]	1.4%
Vanhaesebrouck, et. al	Belgium	1999−2000	175	0.45	[0.37; 0.52]	1.3%
Waal, et. al	Netherlands	2007	144	0.24	[0.18; 0.32]	1.3%
Walsh, et. al	USA, CNN, VON	2001−2003	75974	0.29	[0.28; 0.29]	1.5%
Weber, et. al	Austria	1999−2001	321	0.30	[0.25; 0.35]	1.4%
**Random effects model**				**0.21**	**[0.19; 0.24]**	**100.0%**

Heterogeneity: I^2 = 99%, τ^2 = 0.0144, p = 0

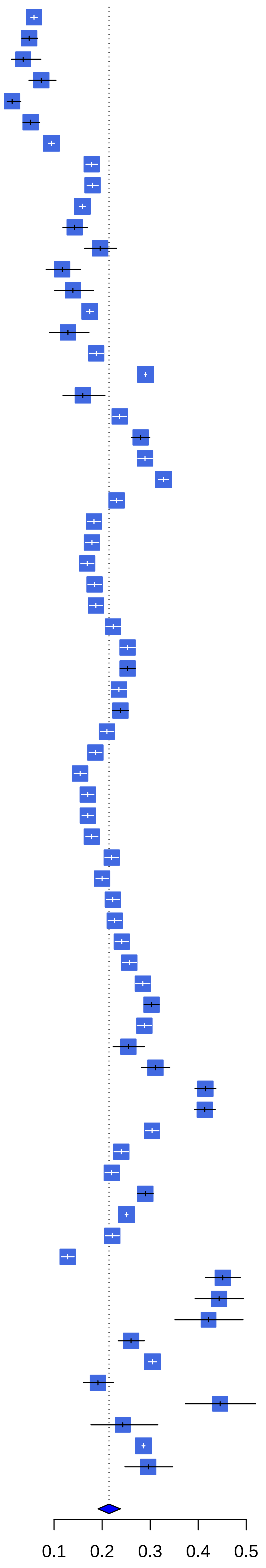

Supplement: Supplementary file 3 — Supplementary Material 3 [file 12931_2024_2850_MOESM3_ESM.pdf]
