# Supplementary material for: Rates of bronchopulmonary dysplasia in very low birth weight neonates: a systematic review and meta-analysis
Source: Respir Res. 2024 May 24;25:219. doi: 10.1186/s12931-024-02850-x (PMC11127341; doi:10.1186/s12931-024-02850-x)

Study	Country	Year	Sample	Proportion	95%−CI	Weight
Kusuda, et. al	Japan	2003	2145	0.28	[0.26; 0.30]	12.0%
Network21, et. al	Australia & New Zealand	2015	3449	0.26	[0.24; 0.27]	14.5%
Network25, et. al	Canada	2004	687	0.25	[0.22; 0.29]	6.1%
Network30, et. al	Japan	2016	3030	0.24	[0.22; 0.26]	13.9%
Network6, et. al	Australia & New Zealand	2000	2972	0.25	[0.24; 0.27]	13.8%
Network7, et. al	Australia & New Zealand	2001	2924	0.25	[0.24; 0.27]	13.7%
Network9, et. al	Australia & New Zealand	2003	2607	0.24	[0.22; 0.26]	13.1%
Stevenson, et. al	USA	1993−1994	999	0.26	[0.23; 0.29]	7.9%
Waal, et. al	Netherlands	2007	144	0.24	[0.18; 0.32]	1.6%
Weber, et. al	Austria	1999−2001	321	0.30	[0.25; 0.35]	3.3%
**Random effects model**				**0.25**	**[0.25; 0.26]**	**100.0%**

Heterogeneity: I^2 = 49%, τ^2 = 0.0001, p = 0.04

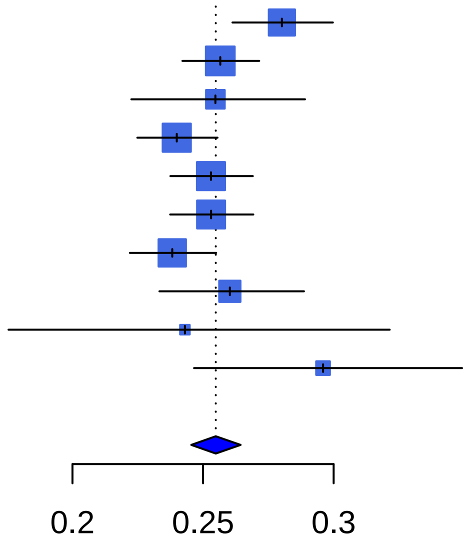

Supplement: Supplementary file 5 — Supplementary Material 5 [file 12931_2024_2850_MOESM5_ESM.pdf]
