# Supplementary material for: Rates of bronchopulmonary dysplasia in very low birth weight neonates: a systematic review and meta-analysis
Source: Respir Res. 2024 May 24;25:219. doi: 10.1186/s12931-024-02850-x (PMC11127341; doi:10.1186/s12931-024-02850-x)
Supplement: Supplementary file 6 — Supplementary Material 6 [file 12931_2024_2850_MOESM6_ESM.docx]

| **Author** | **Death Rate**  **(%)** |
| --- | --- |
| Álvarez-Fuente | 11.7 |
| Ancel | 0 |
| Bevilacqua | 23.9 |
| Bonamy | 0 |
| Chen | 15.6 |
| Choi | 19 |
| Fanaroff | 19 |
| Fanaroff | 15 |
| Fortmann | 3.5 |
| Gortner | 8.4 |
| Grandi | 0 |
| Grisaru-Granovsky | 0 |
| Guimarães | 17.2 |
| Guinsberg | 30 |
| Hentschel | 0 |
| Horbar | 12.5 |
| Kamper | 0 |
| Koc | 0 |
| Kong | 5.5 |
| Kusuda | 11 |
| Lee | 13.2 |
| Lee | 14 |
| Lemons | 16 |
| Marret | 0 |
| Murphy | 16.7 |
| Persson | 8.2 |
| Qiu | 7.6 |
| Rodrigo | Unable to calculate |
| Rutkowska | 0 |
| Sasaki | 7.8 |
| Skromme | 0 |
| Stensvold | 0 |
| Stevenson | 17.5 |
| Su | 8.0 |
| Toome | 0 |
| Tsou | 0 |
| Tyson | 14.1 |
| Vanhaesebrouck | 0 |
| Waal | 0 |
| Walsh | 12.5 |
| Watson | 4.8 |
| Weber | 0 |
