# Supplementary material for: Rates of bronchopulmonary dysplasia in very low birth weight neonates: a systematic review and meta-analysis
Source: Respir Res. 2024 May 24;25:219. doi: 10.1186/s12931-024-02850-x (PMC11127341; doi:10.1186/s12931-024-02850-x)
Supplement: Supplementary file 10 — Supplementary Material 10 [file 12931_2024_2850_MOESM10_ESM.pptx]

## Slide 1
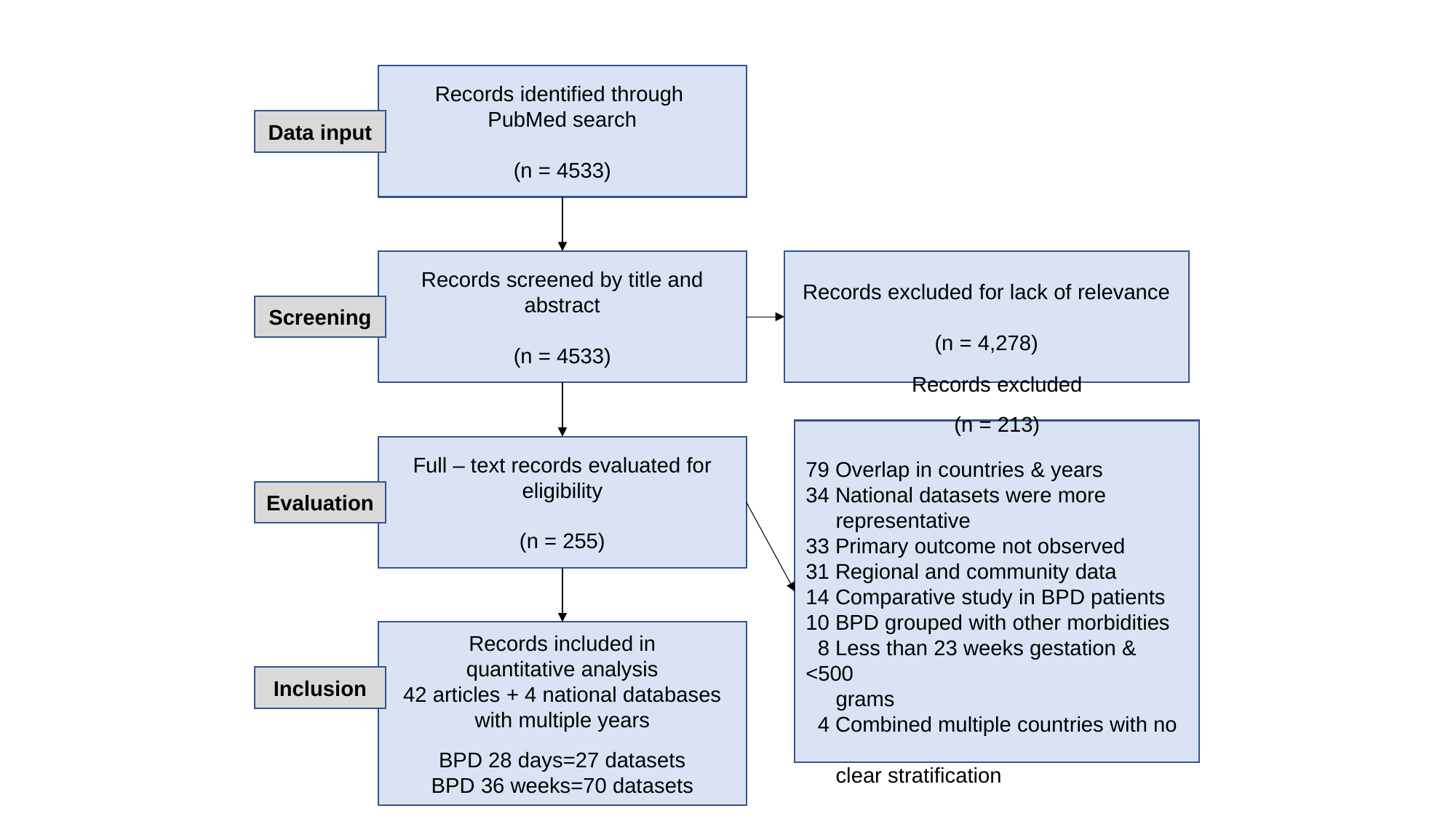

Data input
Records identified through
PubMed search
(n = 4533)
Screening
Records screened by title and abstract
(n = 4533)
Records excluded for lack of relevance
(n = 4,278)
Records excluded
(n = 213)
79 Overlap in countries & years
34 National datasets were more
 representative
33 Primary outcome not observed
31 Regional and community data
14 Comparative study in BPD patients
10 BPD grouped with other morbidities
 8 Less than 23 weeks gestation & <500
 grams
 4 Combined multiple countries with no
 clear stratification
Evaluation
Full – text records evaluated for eligibility
(n = 255)
Inclusion
Records included in
quantitative analysis
42 articles + 4 national databases with multiple years
BPD 28 days=27 datasets
BPD 36 weeks=70 datasets
